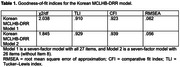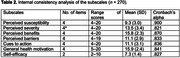# Psychometric Properties of the Korean Version of the Motivation to Change Lifestyle and Health Behaviors for Dementia Risk Reduction Scale in Middle‐Aged and Older Adults

**DOI:** 10.1002/alz.094639

**Published:** 2025-01-09

**Authors:** Hyunseo An, Sohyeon Yun, Inhye Kim, Jiwon Shin, Hyun Yang, Hae Yean Park

**Affiliations:** ^1^ Graduate School, Yonsei University, Wonju, Heungup‐meon Korea, Republic of (South); ^2^ Graduate School, Yonsei University, Wonju, Wonju Korea, Republic of (South); ^3^ College of Software and Digital Healthcare Convergence, Yonsei University, Wonju, Heungup‐meon Korea, Republic of (South)

## Abstract

**Background:**

Despite Korea’s efforts to promote dementia‐prevention lifestyles and health behaviors, adherence among older adults is low. This underscores the importance of identifying factors, “beliefs, and attitudes” that influence lifestyle and health behavior changes to reduce dementia risk. The effectiveness of the scale used to measure these changes has been proven in different cultural contexts, but its suitability for the Korean population has yet to be investigated. Therefore, this study aimed to translate and validate the Motivation to Change Lifestyle and Health Behaviors for Dementia Risk Reduction (MCLHB‐DRR) scale in Korean middle‐aged and older adults.

**Method:**

This study recruited a community‐living sample of Korean adults aged 50 years and older from an online panel. Content validity and construct validity were examined to determine the scale’s validity. Internal consistency and test‐retest reliability were examined to determine its reliability. Test‐retest reliability was evaluated by readministering the MCLHB‐DRR scale to 50 individuals within three weeks of the initial application.

**Result:**

A total of 270 participants completed the questionnaire. Content validity was determined by professors and researchers in occupational therapy. Item 8 was removed from the Korean version after most experts agreed that it did not align with Korean cultural perceptions of dementia’s perceived severity. The confirmatory factor analysis of the 26‐item scale showed a good model fit (χ2/df = 1.845, comparative fit index = 0.939, Tucker–Lewis index = 0.929, root mean square error of approximation = 0.056). Cronbach’s alpha values were .821–.900, indicating good internal consistency. No significant test‐retest score differences were identified between the groups.

**Conclusion:**

These results indicated that the MCLHB‐DRR scale is valid and reliable for the Korean population to assess beliefs and attitudes toward lifestyle changes to reduce dementia risk. Its validation in the Korean context aids in developing lifestyle interventions for dementia prevention and provides foundational data for comparing lifestyle change motivations across countries.